# The Wnt Pathway Controls Cell Death Engulfment, Spindle Orientation, and Migration through CED-10/Rac

**DOI:** 10.1371/journal.pbio.1000297

**Published:** 2010-02-02

**Authors:** Juan Cabello, Lukas J. Neukomm, Ufuk Günesdogan, Katharina Burkart, Steve J. Charette, Günter Lochnit, Michael O. Hengartner, Ralf Schnabel

**Affiliations:** 1 Technische Universität Carolo Wilhelmina Braunschweig, Institut für Genetik, Braunschweig, Germany; 2 Instituto de Biología Molecular y Celular del Cáncer, Centro de Investigación del Cáncer, Universidad de Salamanca-CSIC, Campus Universitario Miguel de Unamuno s/n, Salamanca, Spain; 3 Institute of Molecular Biology, University of Zurich, Zurich, Switzerland; 4 Biochemisches Institut, Universität Gießen, Gießen, Germany; Dartmouth Medical School, United States of America

## Abstract

Wnt signalling pathways have extremely diverse functions in animals, including induction of cell fates or tumours, guidance of cell movements during gastrulation, and the induction of cell polarity. Wnt can induce polar changes in cellular morphology by a remodelling of the cytoskeleton. However, how activation of the Frizzled receptor induces cytoskeleton rearrangement is not well understood. We show, by an in depth 4-D microscopy analysis, that the *Caenorhabditis elegans* Wnt pathway signals to CED-10/Rac via two separate branches to regulate modulation of the cytoskeleton in different cellular situations. Apoptotic cell clearance and migration of the distal tip cell require the MOM-5/Fz receptor, GSK-3 kinase, and APC/APR-1, which activate the CED-2/5/12 branch of the engulfment machinery. MOM-5 (Frizzled) thus can function as an engulfment receptor in *C. elegans*. Our epistatic analyses also suggest that the two partially redundant signalling pathways defined earlier for engulfment may act in a single pathway in early embryos. By contrast, rearrangement of mitotic spindles requires the MOM-5/Fz receptor, GSK-3 kinase, and β-catenins, but not the downstream factors LIT-1/NLK or POP-1/Tcf. Taken together, our results indicate that in multiple developmental processes, CED-10/Rac can link polar signals mediated by the Wnt pathway to rearrangements of the cytoskeleton.

## Abstract 2

Specificity in Wnt-mediated developmental processes, such as directional cell cleavage, migration, and engulfment of dead cells in *C. elegans*, arises from the use of distinct Wnt pathway signalling modules.

## Author Summary

During development, processes such as cell division, fate determination, migration, and removal of dead cells occur in a directional (i.e., polar) manner. For example, cell divisions in the early embryo often occur in a directional manner to maintain specific cell–cell interactions. Later, during organ formation, tissues may be shaped through polar cell migration. During apoptosis, a neighbouring cell engulfing a cell corpse utilizes polar positional information to deform its cytoskeleton and migrate around the corpse. Wnt pathway signalling is a common mechanism by which cells establish polarity during development, but how a single Wnt signal is translated into different outcomes in different cellular or developmental contexts is not clear. Our data on *C. elegans* suggest that during diverse directional events—cell engulfment, mitotic spindle movement, and cell migration—different Wnt ligands or a specific signal from a cell corpse signals to the Wnt receptor Frizzled (MOM-5 in *C. elegans*) and on to the downstream factors CED-10/Rac. But specificity of the Wnt signal is mediated by the use of distinct intermediate signal transduction pathways. Our discovery that this pathway is also used for engulfment of cell corpses led us to propose that the two parallel pathways, originally described for engulfment of corpses, are indeed parts of one pathway involving Wnt pathway components.

## Introduction

Polar cellular processes are essential during development and maintenance of animals. Cell cleavages and possibly associated cell-fate decisions have to be coordinated. Many cell types also undergo directed cell migrations. Last, but not least, supernumerary or sick cells must be removed by apoptosis, which requires a polar reorganisation of the cytoskeleton in the engulfing cell. Wnt signalling pathways have extremely diverse functions during these processes, including induction of cell fates or tumours, guidance of cell movements during gastrulation, and the induction of cell polarity [1],[2]. Wnt can induce polar changes in cellular morphology by a remodelling of the cytoskeleton [3],[4]. However, how activation of the Fz receptor induces cytoskeleton rearrangement is not well understood. The receptor initiating the engulfment of cell corpses in *C. elegans* remained, despite considerable efforts to define all components required for apoptosis genetically [5], elusive for many years.

Extensive work on apoptosis has defined two partially redundant signalling pathways, which converge at CED-10/Rac to mediate actin cytoskeleton rearrangement [5],[6]. The first is defined by the ABC transporter CED-7/ABCA1, the LRP-like receptor CED-1 and its downstream adaptor CED-6/GULP [7]–[10]. The second pathway is defined by the adaptor protein CED-2/CrkII and the CED-12/ELMO-CED-5/DOCK180 complex, which acts as a GEF to activate CED-10/Rac. Mutations in this second pathway not only result in defects in cell corpse engulfment but also result in aberrant distal tip cell (DTC) migration [7],[11].

We show here that MOM-5 (Frizzled) receptor functions as an engulfment receptor upstream of the CED-2/CrkII-CED-12/ELMO-CED-5/DOCK180 complex. This function was not found until now due to pleiotropic and lethal functions of the responsible MOM-5 (Frizzled) receptor, which has hidden its function in corpse removal.

## Results

### *C. elegans ced-10*/Rac Null Mutants Show a Wnt Pathway–Like Phenotype

**Figure 1:**
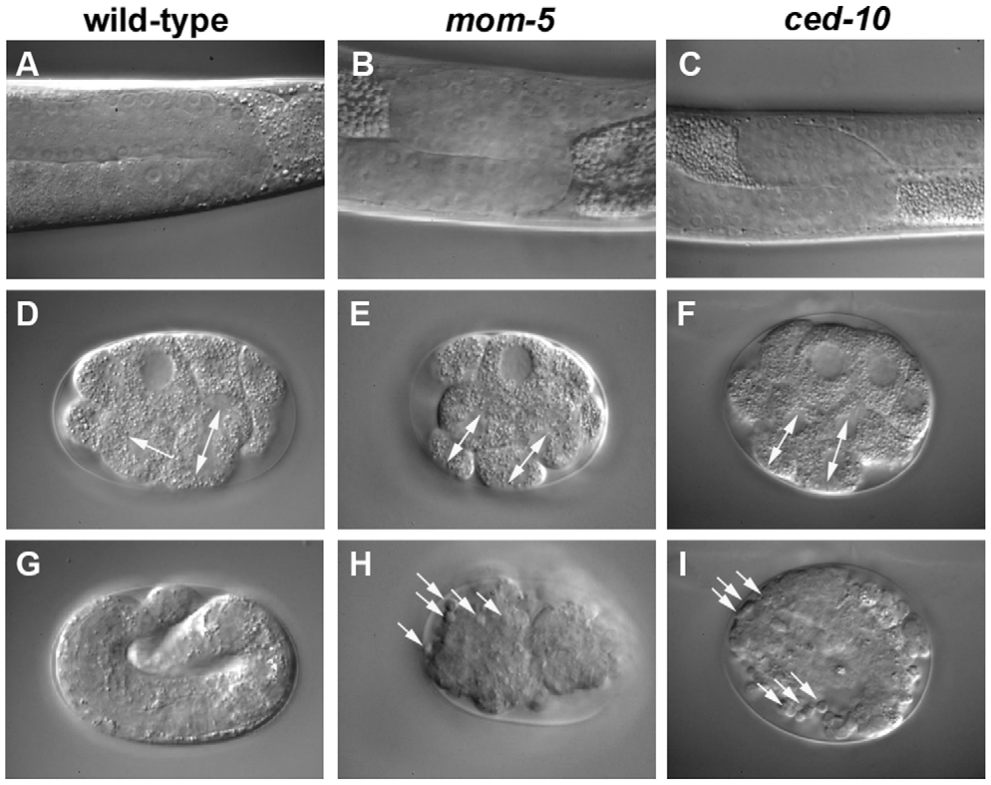
*mom-5* and *ced-10* are required for distal tip cell migration, spindle orientation, and cell corpse engulfment. (A–C) DIC micrographs of gonads of wild-type (A), *mom-5(zu193)* (B), and *ced-10(t1875)* (C) adult hermaphrodites. In the homozygous mutant, maternally rescued animals, the distal arms of the gonad do not migrate back towards the vulva, but loop and migrate into the anterior or posterior of the body. (D–I) DIC micrographs of embryos from wild-type hermaphrodites (D and G) and from hermaphrodites homozygously mutant for *mom-5* (E and H) or *ced-10* (F and I). (D–F) show eight-cell embryos just dividing into the 12-cell stage. The arrows indicate the divisions of the ABar (left) and ABpr blastomeres. In the mutant embryos, ABar does not cleave perpendicular but in parallel to ABpr since the spindle was not turned prior to the cleavage. In contrast to the wild-type embryo (G), the mutant embryos (H and I) accumulate cell corpses since these are not engulfed.

**Figure 2:**
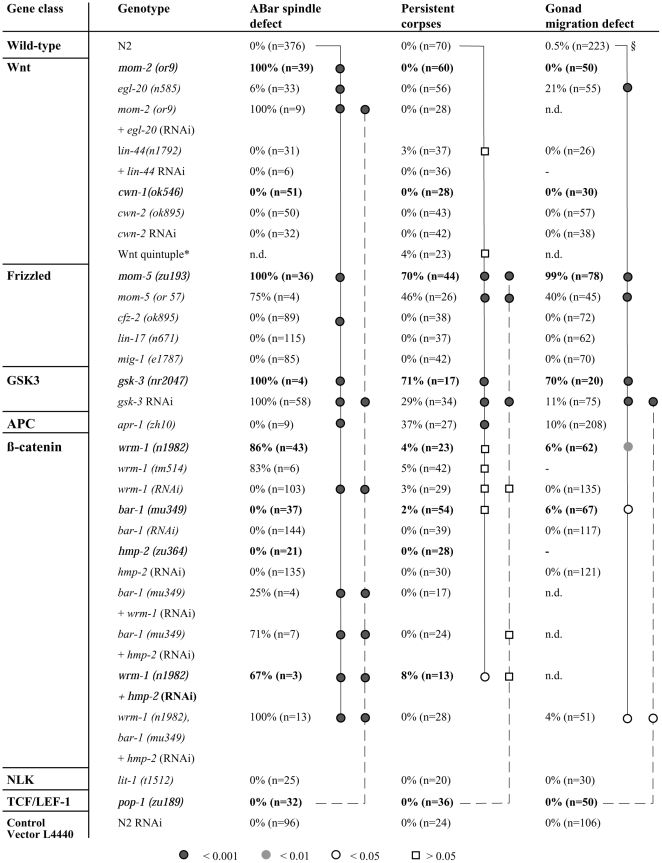
The Wnt pathway affects spindle orientation, engulfment of cell corpses, and gonadal migration. The gonadal migration was scored in the maternally rescued homozygous mothers *(z−m+)*. In some cases, RNAi was used either to explore whether phenotypes can be enhanced further or to avoid construction of multiple mutants. All but the RNAi for *lin-44* were carried out by feeding. After an injection, the gonadal migration cannot be scored (−). The spindle defect and the persistent cell corpses were scored in embryos from such mothers as described in Materials and Methods. As controls, (N2 + L4440 RNAi) was used for the RNAi feeding experiments, and the empty vector L4440 for the injections. (*) Embryos from Wnt quintuple mutant (*lin-44*; *cwn-1*; *egl-20 cwn-2*; *mom-2*) hermaphrodites derived from the strain HS1680 (see Materials and Methods) were analysed to assess possibly redundant functions of the ligands. (§) We found one looping gonad in 223 gonadal arms of wild-type animals. Although we generally rounded the numbers to the full percent, we rounded on this occasion not from 0.49% to 0%, but to 0.5%. The significance of results was tested using the Fischer exact test since it allows—in contrast to the paired chi square test—to test against zero positive events. Statistical tests are shown graphically on vertical lines. The horizontal lines at the end identify the reference genotype (or RNAi). If the symbol next to a genotype (or RNAi) on the vertical line is a circle, then the difference is significant. A square indicates that there is no significant difference. The degree of statistical significance is indicated by the shade of grey used for the symbol.

**Figure 3:**
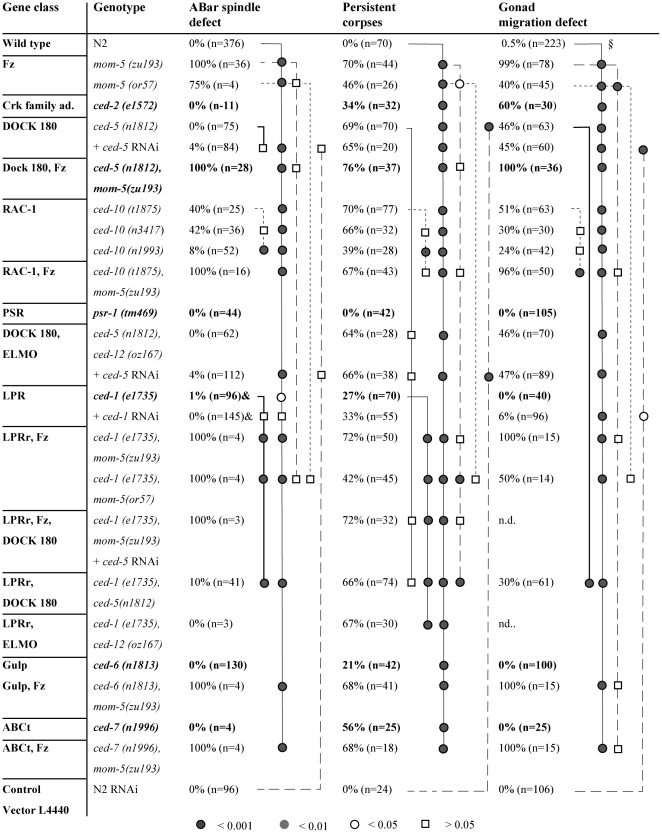
Interactions between Wnt and cell corpse engulfment pathways. For details, see Figure 2. The quality of genetic epistatic analysis depends on the availability of null mutations and on the precision with which the phenotypes can be assessed. Some of the mutations of the *ced* genes are nonsense alleles and could be thus very strong or even null alleles. On the other hand, all alleles were isolated as viable mutants, which could mask potential essential functions like the proper orientation of cell cleavage during embryogenesis. That a null allele of *ced-10* was only isolated in a screen for embryonic lethal mutants exemplifies the problem [6]. To potentially lower residual gene activity, we applied RNA interference to some mutants and used 4-D microscopy to evaluate the direction of the cleavage of ABar and to quantify the number of apoptotic cell corpses that are not engulfed. (&) Although the single event in the only mutant embryos is significant, the single event is statistically insignificant if one also considers the embryos additionally treated with RNAi.

During our 4-D microscope [12] analysis of maternal-effect lethal mutations on linkage group IV of *C. elegans*, we noticed a striking set of pleiotropic defects in embryos laid by mothers homozygous for the *t1875* mutation, which we showed to be a null allele of *ced-10*/Rac [6] (Figure 1). The first visible phenotype seen by 4-D microscopy is that the spindle poles of blastomeres EMS and ABar are not rotated 90° as in wild-type embryos (Figure 2). As a result, ABarp, instead of ABara, contacts the MS blastomere in the12-cell–stage embryos. MS then does not induce the right ABara fate in the anterior daughter of ABar, but induces the left ABalp fate in the posterior daughter of the ABar blastomere. This disrupts the normal left–right asymmetry in these embryos, which as a result fail to undergo morphogenesis. In addition to this specific cell-fate transformation, second-generation (*m−z−*) (maternal and zygotic defective) *t1875* embryos contain a large number of apoptotic cell corpses (Figure 1) due to a defect in apoptotic cell clearance [6]. Neither defect is apparent in first-generation (*m+z−*) *t1875* embryos, likely due to maternal rescue. First-generation *t1875* mutants do, however, show a number of postembryonic defects, including looping gonads due to abnormal migration of the DTCs [7]. All three defects are also observed in other *ced-10* alleles, including the deletion allele *n3417* (Figure 3). The spindle defect of *ced-10* animals suggests that CED-10/Rac is not only required for cell migration and the initiation of engulfment of cell corpses, but also for the rotation of the mitotic spindle in EMS and ABar [13]–[15]. A similar defect in EMS and ABar spindle rotation has previously been observed in embryos lacking the *C. elegans frizzled* homologue *mom-5* [16],[17] (Figures 1 and 2). Activation of MOM-5 via the Wnt family member MOM-2 is thought to regulate spindle positioning by controlling actin cytoskeletal rearrangement. Interestingly, MOM-5 signalling to the cytoskeleton requires GSK-3, but not the transcription factor POP-1/TCF [4]. Our observations thus suggest that CED-10/Rac might be a novel component in Wnt signalling, linking MOM-5/Fz and GSK-3 to cytoskeletal rearrangement in spindle positioning in *C. elegans*.

### *mom-5* Mutants Show Defects in Apoptotic Corpse Engulfment and Gonadal Distal Tip Cell Migration

**Figure 4:**
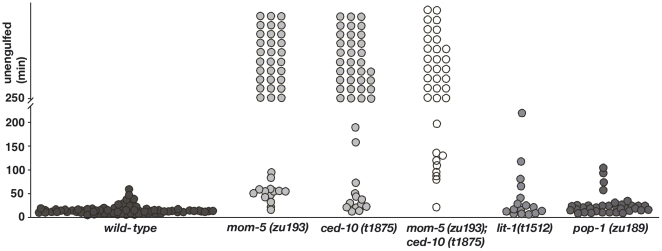
MOM-5 affects the engulfment of cell corpses in the embryo. The figure shows the kinetics of the engulfment of corpses formed during the first wave of cell death in the embryo. The genotypes of the mothers are indicated. Dots are placed on the graph at the time points when the corpses were engulfed after their appearance. Corpses that were not engulfed after 4 h are considered not to be engulfed and are piled up merely to show the number.

To determine whether Wnt signalling might also regulate other Rac-dependent processes, we performed an in-depth phenotypic analysis of *mom-5* mutants. A 4-D analysis of the *mom-5* null allele *zu193* [16] showed that 70% of the cell corpses generated during the first wave of embryonic cell deaths failed to be engulfed. A weaker allele, *mom-5(or57)* [17] yielded 46% persistent corpses (Figure 2). Mutant embryos develop with normal rates, thus the failure to engulf corpses is not caused by a general “sickness” or arrest of embryos (Figure S1). The timing of engulfment in *mom-5* is shown in Figure 4. These results suggest that MOM-5/Fz function is required for efficient cell corpse clearance in *C. elegans* embryos. Finally, we also found a high frequency (99%) of gonad migration defects in (maternally rescued) *mom-5(zu193)m+z−* hermaphrodites (Figures 1 and 2), suggesting that MOM-5/Fz might also function together with CED-10/Rac in this process.

### Wnt Pathway Components Contribute to Spindle Positioning, Cell Corpse Clearance, and Distal Tip Cell Migration

Multiple distinct Wnt pathways, often labelled as “canonical” and “noncanonical,” have been described both in worms and other species (reviewed in [18]). These various pathways use distinct, but partially overlapping, sets of proteins. To determine whether other Wnt pathway components might participate with MOM-5 in spindle positioning, cell corpse clearance, and DTC migration, we analyzed various mutants for defects in these three processes.

Consistent with previously published data, ABar spindle positioning required MOM-2/Wnt and GSK-3 function, but not WRM-1/β-catenin or POP-1/TCF (Figure 2; [4]), suggestive of a noncanonical Wnt signalling pathway. These last elements (WRM-1, POP-1) have been described to indirectly regulate the timing of spindle rotation [19]. To our surprise, however, we found that ABar positioning also required the β-catenin WRM-1 (and to a lesser extent also the other β-catenin homologues HMP-2 and BAR-1; Figure 2), but not the downstream component LIT-1/NLK or POP-1/TCF. These results suggest that ABar spindle positioning is regulated via a novel type of Wnt signalling in which WRM-1/β-catenin signals through an unknown pathway to CED-10.

We found that a similar, but distinct pathway likely acts in engulfment and DTC migration. Animals lacking MOM-5/Fz, GSK-3, or APR-1 showed both persistent cell corpses and misshapen gonads, whereas loss of β-catenins, LIT-1/NLK, or POP-1/TCF did not result in any significant engulfment or DTC migration defect (Figures 2 and 4). MOM-5, LIT-1, and POP-1 influence many cell-fate decisions during embryonic development. The fact that *pop-1* and *lit-1* mutants do not show persistent cell corpses suggests that the engulfment defect of *mom-5* mutants is specific and not a secondary consequence of cell-fate transformations.

Five *C. elegans* WNT ligands (MOM-2, EGL-20, LIN-44, CWN-1, and CWN-2) have been reported to signal to Frizzled [20]. Consistent with previous reports [16],[17], we found that MOM-2 plays an essential function in promoting ABar spindle orientation. We also observed a spindle orientation defect in a small fraction (6%) of *egl-20(n585)* mutant embryos, suggesting that EGL-20 may to some extent act together with MOM-2 on the Frizzled receptor. Consistent with published data [21], only *egl-20(n585)* mutants showed any defect in gonadal migration (21%; Figure 2). Finally, none of the investigated five WNT homologues had any significant effect on cell corpse engulfment when inactivated individually. Even a quintuple inactivation of the Wnt family members had no significant effect (Figure 2). This suggests that these genes also do not act redundantly during engulfment and that (an)other signal(s) is used to engage the engulfment machinery.

### Wnt Pathway Signals to CED-10 to Regulate Clearance of Cell Corpses

**Figure 5:**
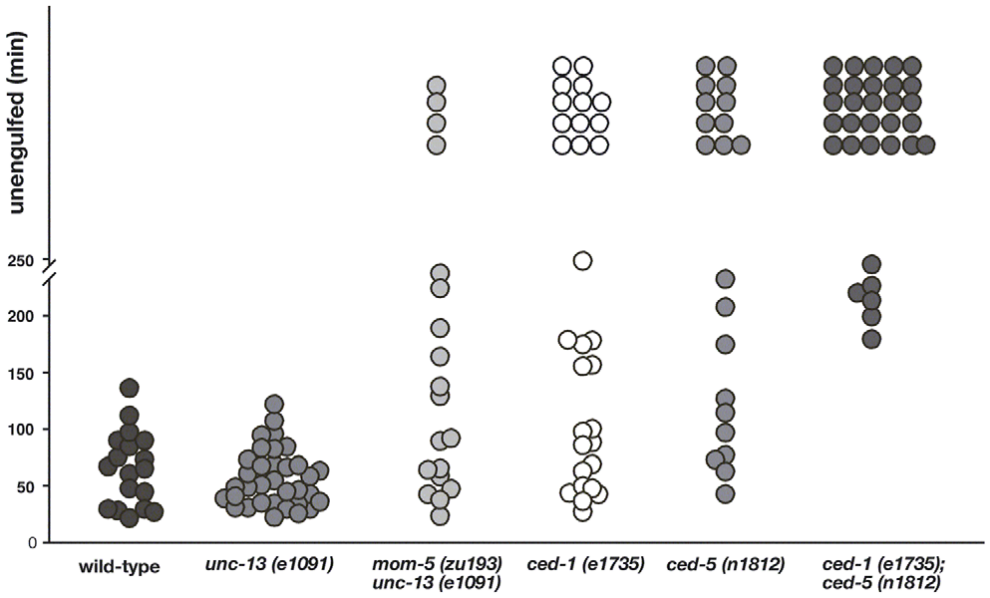
MOM-5 affects the engulfment of cell corpses in the gonad. The figure shows the kinetics of the engulfment of corpses in the gonad. See legend to Figure 4 for details.

To determine how the Wnt pathway links to CED-10/Rac, we undertook a series of single-mutant and double-mutant analyses using 4-D microscopy, which is, as shown earlier [6],[22], well suited to assess birth, death, engulfment, and degradation of a programmed cell death (Figures 3 and 4). Approximately 70% of cells dying in the first wave of embryonic apoptosis failed to be engulfed in *mom-5*, *ced-5*, or *ced-10* single mutants, whereas *ced-1*, *ced-7*, or *ced-6* single mutants showed a weaker engulfment defect. Surprisingly, the engulfment defect of double mutants between any two of these genes was never stronger than the stronger of the two single mutants. In other words, double mutants between any two of these genes did not cause any further increase in persistent cell corpse number. The most parsimonious explanation for our observations is that MOM-5 acts together with the engulfment *ced* genes in a single, common pathway to mediate clearance of the first wave of embryonic cell deaths in the embryo. This result is surprising given the well-established partial redundancy between the *ced-2*/*5*/*12* and *ced-1*/*6*/*7* pathways in the removal of both somatic and germline corpses [5],[6],[23]. The redundancy in the gonad we also observed here (Figure 5, Table S1).

We performed a much longer time-course experiment, counting the number of cell corpses present through embryonic and larval development in *ced-1* and *ced-5* single and double engulfment mutants (Figure S2). Interestingly, we found that the additive engulfment defect in *ced-1*; *ced-5* double mutants is quite pronounced at later time points (e.g., mid-L1, 1,200 min postfertilization), but rather mild during early embryogenesis (e.g., comma stage, 400 min), consistent with our 4-D lineage data (Figure 3). This suggests that different processes might promote the clearance of fresh corpses and the late clearance of persistence corpses (scavenger engulfment).

To determine whether Wnt signalling also promotes clearance of germ cell corpses, we counted apoptotic cells in the germline of maternally rescued (*m+z−*) *mom*-5 mutants. Unlike *ced-1* and *ced-5*, *mom-5(m+z−)* animals did not show an increased number of germ cell corpses. However, these animals have a highly reduced number of germ cells, which might affect the extent of germ cell apoptosis. To bypass this problem, we directly measured germ cell corpse persistence via 4-D video recording. In this more sensitive assay, we found that nearly 40% of germ cell corpses in *mom-5(m+z−)* animals persisted for over 3 h (Table S1, Figure 5), compared to 57% and 67% of corpses for *ced-1* and *ced-5* mutants, respectively. These results suggest that *mom-5* functions also in the clearance of apoptotic germ cells.

### MOM-5 Acts through CED-5 and CED-10 to Control Engulfment of Apoptotic Corpses and Distal Tip Cells Migration

**Figure 6:**
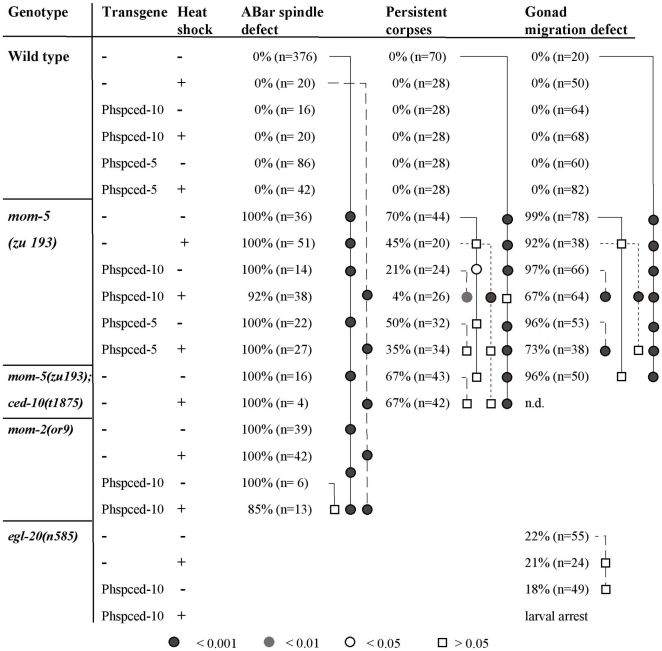
Overexpression of CED-10/Rac bypasses loss of MOM-5/Fz. For details, see legend to Figure 2.

We tested whether overexpression of CED-5 or CED-10 bypasses the requirement for MOM-5 (Figure 3). In a *mom-5(zu193)* mutant background, the overexpression of CED-10 reduced the persistent cell corpses by more than 90% when compared to the heat-shock control. Heat shock per se also improved engulfment in the mutant. Interestingly, this improvement depends on CED-10/Rac, since it does not occur in the double mutant. Overexpression of CED-5 was also able to weakly suppress the *mom-5* defect (Figure 6).

We also analysed how MOM-5 controls DTC migration and spindle polarity. Because *mom-5* single mutants already show an almost complete migration defect, we could not use double-mutant analysis to determine whether MOM-5 signals to the CED-2/5/10 pathway. However, overexpression of both CED-5 and CED-10 significantly suppressed the *mom-5* DTC migration defect (Figure 6), suggesting that this is indeed the case. Consistent with previous studies [10], we found that the CED-1/6/7 pathway appears to play at most a minor role, if any, in this process. It appears to be specific for the engulfment of corpses (Figure 3).

### Phosphorylated APR-1/APC Interacts with CED-2/CrkII in a Yeast-Two-Hybrid Assay

**Figure 7:**
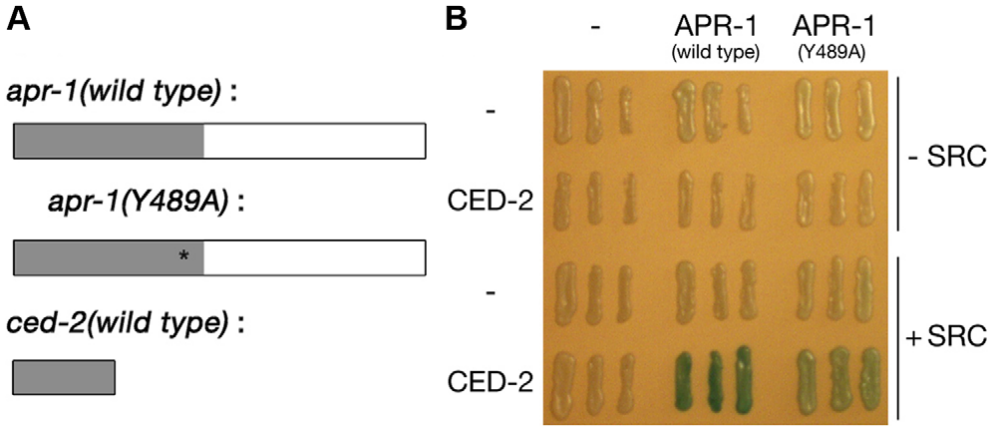
APR-1 is involved in engulfment signalling. (A) Shown are full-length cDNAs (white boxes) and used fragments (grey shading) for yeast two-hybrid. *apr-1(wild type)* and *apr-1(Y489A)*: amino acids 1–545 of a total of 1,188. *ced-2(wild type)*: full-length cDNA. (B) Three individual clones were used to test interaction, either with or without a constitutive active *Src* kinase in the background (+Src and −Src, respectively).

CED-2, the *C. elegans* homologue of mammalian CrkII, is an adapter protein containing an N-terminal phospho-tyrosine–binding SH2 and two C-terminal SH3 domains. By analogy with its mammalian homologue, CED-2 has been proposed to bind to upstream activating signalling molecules via its SH2 domain, and relay this information to CED-5 via its first SH3 domain (Figure 7). To identify new inputs into the engulfment signalling pathway, we performed a yeast two-hybrid screen for proteins that interact with the SH2 domain of CED-2 in a phospho-tyrosine–dependent manner (see Materials and Methods). Interestingly, one of the two strongest phosphorylation-dependent interactors identified in this screen was APR-1, the *C. elegans* APC homologue (Figure 7). In *Drosophila* and mammals, APC is known to physically interact with GSK-3 to regulate β-catenin phosphorylation. The physical interaction between APR-1 and CED-2 might thus allow the cell to direct GSK-3 signalling towards the Rac pathway rather than towards β-catenin. Consistent with this hypothesis, we found that loss of *apr-1* function results in a persistent cell corpse phenotype similar in strength to that of *ced-2* mutants (Figure 3). Despite extensive proteomics efforts, we were unable to identify any interaction between CED-2 and APR-1 in vivo (Text S1). This result is not surprising, given that we expect the interaction between phospho-APR-1 and CED-2 to only be transient and to occur only in a few cells at any time.

## Discussion

Based on the results presented here, we propose that MOM-5 (Frizzled) functions as a (co)receptor for the engulfment of apoptotic cell corpses. As expected from our genetic evidence, the receptor is expressed in all cells [24] and clusters around cell corpses (Figure S3). Extensive genetic analyses of the removal of apoptotic corpses during development and in germline have uncovered two pathways defined by the components CED-1/6/7 and CED-2/5/12 [6],[10]. The first may well define a receptor system to recognise the corpse; however, its link to the cytoskeleton, which has to be modulated for engulfment, remained elusive despite considerable efforts. The second pathway defined by CED-2/5/12 will modulate the cytoskeleton. But, how this pathway is activated by the presence of a corpse is hitherto unknown. To resolve the problem, we propose here that a third pathway, the Wnt pathway, links the first two pathways into a single system sensing and engulfing cell corpses, at least during the first wave of cell death in the embryos.

Our analysis of mutants in components of the Wnt pathway presented here reveals new functions for the pathway. It is involved not only in fate specification and spindle orientation, but also in engulfment of apoptotic corpses and migration of the gonadal DTCs. Since the last functions have been already described for *ced* mutants, we tested the hypothesis that the Wnt and *ced* pathways may interact. The epistatic and bypass experiments indeed indicate that MOM-5/Fz activates CED-10/Rac through the GEF complex CED-2, CED-5, CED-12. The in-depth 4-D analysis of the first round of embryonic apoptosis showed, to our own surprise no additive effect between any of the *ced* or Wnt pathway mutants, which is classically taken as indication that genes are functioning in one pathway. This contrasts the earlier notion of “two separate pathways” derived from assays in which apoptotic corpses in the gonad or in the head of the larva are counted. Kinchen et al. (2005) already showed using a null allele of *ced-10*, that there are no additive effects with mutants in the other *ced* genes when the counting assays are used [6]. Thus a potential problem in the earlier analyses could be that null alleles were not always used. Besides this technical problem, at least two hypotheses could also explain the difference. It is possible that the first wave of embryonic cell deaths is removed via a signalling pathway that is distinct from the one used later in development, although the earlier observations by Kinchen et al. (2005) just discussed suggest that all embryonic corpses may be removed by a single pathway [6]. Still, 4-D analysis of cells and global cell corpse counts could measure different phenomena. Our long-term analysis of persistent corpses through embryonic and larva development shows, indeed, that the additive engulfment defect in *ced-1*; *ced-5* double mutants is quite pronounced at later time points, but rather mild during early embryogenesis. This suggests that different processes might promote the clearance of fresh corpses and the late clearance of persistence corpses (scavenger engulfment).

Regarding the ABar spindle orientation, where both *mom-5* and *ced-10* had a significant effect on ABar cleavage (Figures 2 and 3), neither *apr-1* nor any of the other *ced* genes showed a strong defect, suggesting that the link to CED-10 may not occur through CED-2/5/12 as is the case for engulfment and DTC migration. Consistent with this hypothesis, we found that the β-catenin WRM-1 (and to a minor extent, BAR-1 and HMP-2) is required for spindle orientation, but not for cell corpse clearance or DTC migration (Figure 2). Nevertheless, we did observe spindle defects in a small fraction of *ced-1*; *ced-5* double mutants (Figure 3). We thus cannot exclude that *ced-1* and *ced-5* are still involved—maybe in a partially redundant fashion—in all three cellular processes described here.

Both MOM-5 and CED-1 encode cell surface receptors. Why does clearance of apoptotic cells require both receptors, whereas only MOM-5 is required for DTC migration or spindle orientation? An attractive explanation for these results may be that CED-1 acts as a specific coreceptor for MOM-5 during apoptotic cell recognition. Indeed, the CED-1 homologues Arrow and LRP-5/6 are known to act as a coreceptor for Fz proteins in flies and vertebrates, respectively [25]–[27]. By analogy, we propose that a signal from the dying cell could activate the CED-1/MOM-5 on neighbouring cells, most probably, as argued earlier, without requirement of any WNT ligand. It is possible that phosphatidylserine activates CED-1 instead [28]–[30] and that MOM-5 is used by the receiving cell to polarize its cytoskeleton and activate “migration” around the apoptotic cell. According to this scenario, both *ced-1* or *mom-5* should be epistatic to *ced-10*. As shown here and earlier [6], this is indeed the case.

**Figure 8:**
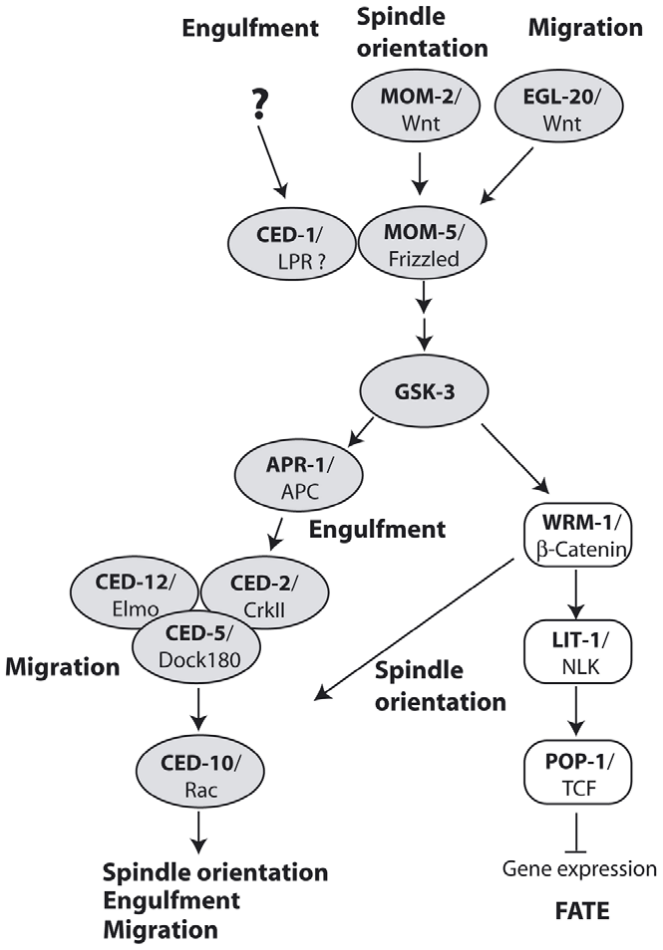
The Wnt pathway and the polarisation of the cytoskeleton in *C. elegans*. Here, we amend the Wnt pathway [4] by showing that GSK-3 and APR-1 are directing apoptotic engulfment and DTC migration by modulating the cytoskeleton through the cell death pathway defined by *ced-5/ced-10*. WRM-1/β-catenin is the branch point to control cell-cleavage direction.

In summary, we show that spindle orientation, engulfment of apoptotic cells, and cell migration depend on a common process of cell cytoskeleton modification, which is under the control of at least two distinct pathways leading from a polar signal through MOM-5/Frizzled to CED-10/Rac (Figure 8). Since the components of the WNT and the apoptotic cell clearance pathway—which we show combine to form a novel pathway for the transduction of cell polarity—are highly conserved from worm to humans, we expect that a similar pathway might contribute to the regulation of cell polarity in mammals also.

## Materials and Methods

### Strains and Genetics

Animals were grown at 25°C as described [31]. The following mutations were used: LG I: *ced-1(e1735)*, *ced-12(oz167)* [32], *dpy-5(e61)*, *Gsk-3(nr2047)* (CGC), *hmp-2(zu364)* [33], *lin-17(n671)*, *lin-44(n1792)*, *mig-1(e1787)*, *mom-5(zu193)*, *mom-5(or57)* [16], *pop-1(zu189)*, *unc-13(e1091)* [34]; LG II: *cwn-1(ok546)* [35]; LG III: *ced-6(n1813)* [36], *ced-7(n1996)* [36], *lit-1(t1512)* [37], *wrm-1(n1982)* (gift from Mello C.), *wrm-1(tm514)* (NBP Japan); LG IV: *egl-20(n585)*, *ced-10(t1875)* [6] *ced-10(n3417)*, *ced-10(n1993)*, *ced-5(n1812)* (CGC), *cwn-2(ok895)* [35], *psr-1(tm469)* [38], *unc-24(e138)*, *unc-42(e270)*; LG V: *cfz-2(ok1201)* (from CGC), *dpy-11(e224)*, *mom-2(or9)* [39], *mom-2 (ne874ts)* [40]; LG X: *bar-1(mu349)* [41] *glp-1(e2144)* [42]. Quintuple Wnt mutant embryos were derived from nonGFP UncEglPsa hermaphrodites segregated by the strain HS1680 (*lin-44(n1792) zdIs5*, *cwn-1(ok546)*, *egl-20(n585) cwn-2(ok895)/nT1 [qIs48]*, *mom-2(ne874ts)/nT1*) [43] at 20°C. Embryos were recorded at 25°C. Hsp::ced-5– and hsp::ced-10–containing plasmids were a gift from H. R. Horvitz. Unless noted otherwise, mutations were previously described [44].

### Analysis of Cell Death by 4-D Microscopy

The 4-D microscopic analysis was carried out at 25°C as described [12]. All data concerning cell death were derived from full recordings starting approximately at the four-cell stage of the embryo. In many cases, lineages were followed until the cell died and then the corpse was followed until it was engulfed or the recording ended. Numbers for statistical purposes were also determined by observing corpses emerging in the 4-D recording. This approach was applied especially to those mutants in the Wnt pathway that cause lineage transformations. During normal embryogenesis, cell death becomes apparent 24±7 min (mean ± standard deviation [s.d.], *n* = 39) after cells are born and engulfment occurs 38±13 min (*n* = 39) later. The latest engulfment we observed during the analysis of the 39 deaths analyzed in normal embryos occurred 83 min after the cell was born and 35 min after the cell died. We also analysed for 31 additional corpses the time between death and engulfment. In *glp-1(e2144)* mutant embryos, we observed the same numbers concerning onset of cell death (26±8; ± s.d., *n* = 17) and engulfment (35±8 min, ± s.d., *n* = 18, both *p*>0.4). The last engulfment occurred 58 min after the cell was born. This cell died 36 min before engulfment. Embryos were recorded for at least 240 min after the first round of cell death occurs in the AB-lineage after the ninth cleavage (ten times longer than what the initiation of engulfment takes normally; three times longer than what the slowest engulfment took that we ever observed in normal embryos). Nevertheless, we cannot exclude that also during normal development, corpses fail to be engulfed. We observed more than 2,400 cell corpses in this work. To get significant numbers for the spindle rotation in ABar, which as opposed to the cell deaths occurs only once per embryo, groups of embryos were recorded until the cleavage occurred. We did not observe an “unspecific” failure of the rotation of the ABar spindle in 376 wild-type embryos. This aberration can be scored unambiguously, and it is therefore highly significant. To get reliable data for the engulfment of cell death in the gonad, we used in addition to the classical procedure [7]—in which the number of refractile corpses are counted 12 h after the L4 larval moulting—also 4-D recordings of gonads starting 12 h after the L4 larval moulting. Worms were paralyzed with levamisol and were mounted under the microscope. Twenty-five focal levels of one gonadal arm were recorded for 8 to 10 h in order to capture every apoptotic event in this time window. Corpses were detected by their refractile shape under DIC optic and followed along in time using SIMI Biocell (see also Table S1).

### Construction of Transgenic Animals

Transgenic strains were constructed by biolistic transformation [45], shooting at N2 worms or *mom-5(zu193)* mutants the plasmids P_hsp_::ced-10 and P_hsp_ced-5 (kind gift of H. R. Horvitz). The pGK10 plasmid (P_serca_::gfp; kind gift of Devgen) was used as selection marker for cotransformation.

### Heat-Shock Experiments

For counting persistent corpses or scoring the ABar spindle direction, adult transgenic animals were shifted once from 15°C to 33°C for 1.5 h, and animals were allowed to recover at 20°C for 5 h before recording of the embryos was started [7]. To analyse the migration of the DTC, synchronised transgenic L1 larvae were shifted from 15°C to 33°C for 1.5 h and incubated at 20°C for 6 h before the next heat shock. The cycles were repeated for 3–4 d until the worms became adults and the gonad was scored.

### RNA Interference

RNA-mediated gene interference (RNAi) was performed by injection [46] or by feeding, as described in [47]. For feeding, synchronised L1 larvae were spotted onto feeding plates and incubated at 15°C until the gonads of adults and embryos of the next generation could be scored. The feeding strains were received from the MRC Geneservice.

### Yeast Two-Hybrid

As a bait, the full-length *ced-2(cDNA)* was cloned 3′ to the LexA DNA binding domain (BD) (LexA-BD-CED-2) in pBTM116-Src vectors (kind gift of L. R. Rohrschneider; [48]). In addition to the expression of the bait protein, this vector allows the expression of the constitutively active Src kinase in yeast. A *C. elegans* cDNA library randomly cloned 3′ to the Gal4 activation domain (AD) was used as prey (Gal4-AD-geneX, pGAD-GH). The yeast two-hybrid screen was performed as described [49] and *apr-1* identified as a potential target: The yeast reporter strain grew on synthetic medium lacking leucine, tryptophan, and histidine (Leu^−^ Trp^−^ His^−^) plates. *apr-1* wild-type and Y489A clones were retested for β-galactosidase activity on Leu^−^ Trp^−^ plates containing X-Gal in the presence or absence of the constitutively active *Src* kinase to determine the phosphotyrosine dependency of the interaction.

## Supporting Information

Figure S1 **Development and viability of** ***mom-5*** **embryos.** The lineage graph (anterior lineages to the left) shows the cleavages of representative AB-derived cells in normal and *mom-2* embryos. The ABar-derived blastomeres are transformed to ABala and ABalp fates in *mom-5* embryos since cell contacts are altered. This causes lineage alterations. Coloured bars indicate the timing of events. Onset of cell death is slightly delayed in *mom-5* mutants. As shown previously, execution of cell deaths depends on the interaction between the prospective cell deaths and its neighbours [6],[22], which may be disturbed in *mom-5* mutants.
(0.39 MB TIF)

Figure S2 **Long-term persistence of apoptotic corpses.** Worms were mounted on 4% agarose pads in a drop of M9 buffer containing 30 mM NaN_3_ or 3–5 mM levimasole and observed by microscopy using a Leica DMRA microscope. Images were taken using an Orca ER camera using Openlab software. Worms were staged as L1s by hypochlorite treatment. Apoptotic cell corpses were identified as highly refractile disks in the germline of identically treated hermaphrodites using Nomarski optics at the time points shown in Table S2.
(0.40 MB TIF)

Figure S3 **Expression of MOM-5 during engulfment of cell corpses in the embryo.** The transgenic line GE4125 shows a late expression of MOM-5::mCherry, mostly in the nervous system. This mosaic expression in a wild-type background can be used to assign the localisation of the receptor to cells surrounding a cell corpse. Since all cells express MOM-5 in the embryo [24], the receptor is also present and even very much concentrated in the shrunken corpse. An example is shown in the top panels. The mother (green arrow [A]) of the corpse (green arrow) shown in (B) (inlet, top right) expresses the transgene (259 min of development). At 304 min, the corpse contains a significant amount of the fusion protein (mCherry fused to MOM-5 to replace the STOP codon) also in the interior. The mother (blue arrow [C]) of the corpse (blue arrow) shown in (D) (inlet, bottom right) does not express any visible amount of the transgene (259 min). This unlabelled corpse migrates to meet cells displaying the fluorescent signal on their surface touching the corpse (304 min). Thus the signal can be unambiguously assigned to its neighbours. The corpse is engulfed at the time the picture was taken by a cell below the corpse. These observations are consistent with our proposal of MOM-5 being involved in the engulfment of cell corpses.
(2.07 MB TIF)

Figure S4 **Functional rescue of the** ***ced-2::tap*** **construct.**
(0.12 MB TIF)

Table S1 **The Wnt pathway affects engulfment of cell corpses in the gonad.** The table shows that the classical method (Refractile corpses [DIC]) for scoring corpses by counting the corpses 12 h after the L4 moult [7] may fail to recognise an engulfment defect after a gene inactivation. This may happen if a reduction of germ cells (Progeny), as observed in *mom-5(zu193)*, causes a reduction of cell corpses occurring in the gonad. If those, however, survive much longer because they are not engulfed, the count by the classical method may be “accidentally” normal. If corpses are followed by 4-D microscopy (Persistent corpses [4-D recording]) the engulfment defect is seen. Corpses in normal gonads are engulfed after 74.8±37.4 min (±s.d. n = 18, low: 31 min, high: 166 min). In *mom-5(zu193)* hermaphrodites that were in addition subjected to an RNAi against MOM-5 to deplete the maternally supplied RNA, the corpses were engulfed after 159.1±126.2 min (±s.d. *n* = 18, low: 32 min, high: 459 min). Considering all cell deaths, the difference is significant (*t*-test *p*<0.02, Mann-Whitney test one-sided *p*<0.05). We scored corpses as not properly engulfed that were not engulfed at a time equal to the wild-type mean +3 s.d. (187 min). The exact timing of events is shown in Figure 5.
(0.04 MB DOC)

Table S2 **List of developmental stages during embryogenesis and larval development analysed in Figure S2.** The time after fertilisation is shown in parentheses.
(0.03 MB DOC)

Text S1 **Biochemical analysis to demonstrate a APR-1 CED-2 interaction in vivo.** A bar graph comparing the rescue of the mutant, Ced, and adult lethal phenotypes is shown in Figure S4.
(0.03 MB DOC)

## Abbreviations

DTC: distal tip cell
RNAi: RNA interference
