# Supplementary figures and images for: The Wnt Pathway Controls Cell Death Engulfment, Spindle Orientation, and Migration through CED-10/Rac
Source: PLoS Biol. 2010 Feb 2;8(2):e1000297. doi: 10.1371/journal.pbio.1000297 (PMC2814829; doi:10.1371/journal.pbio.1000297)

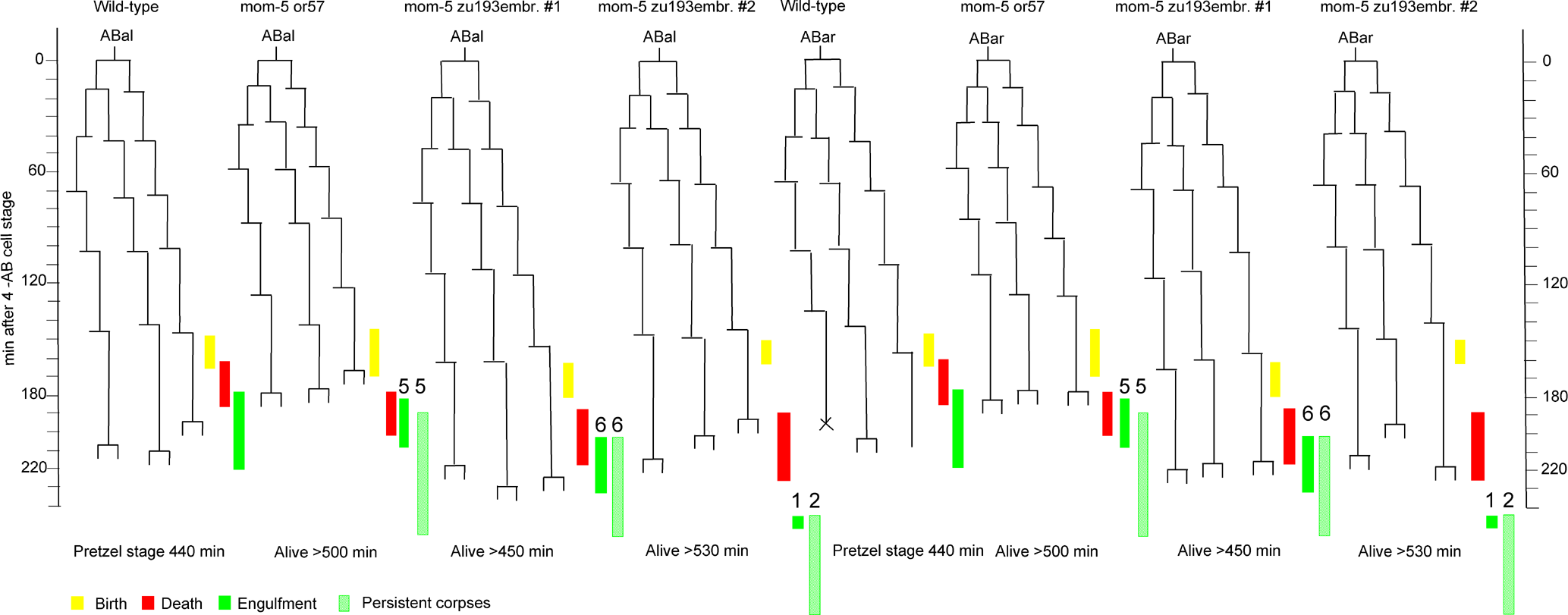

Supplement: Figure S1 — Development and viability of mom-5 embryos. The lineage graph (anterior lineages to the left) shows the cleavages of representative AB-derived cells in normal and mom-2 embryos. The ABar-derived blastomeres are transformed to ABala and ABalp fates in mom-5 embryos since cell contacts are altered. This causes lineage alterations. Coloured bars indicate the timing of events. Onset of cell death is slightly delayed in mom-5 mutants. As shown previously, execution of cell deaths depends on the interaction between the prospective cell deaths and its neighbours [6],[22], which may be disturbed in mom-5 mutants. (0.39 MB TIF) [file pbio.1000297.s001.tif]

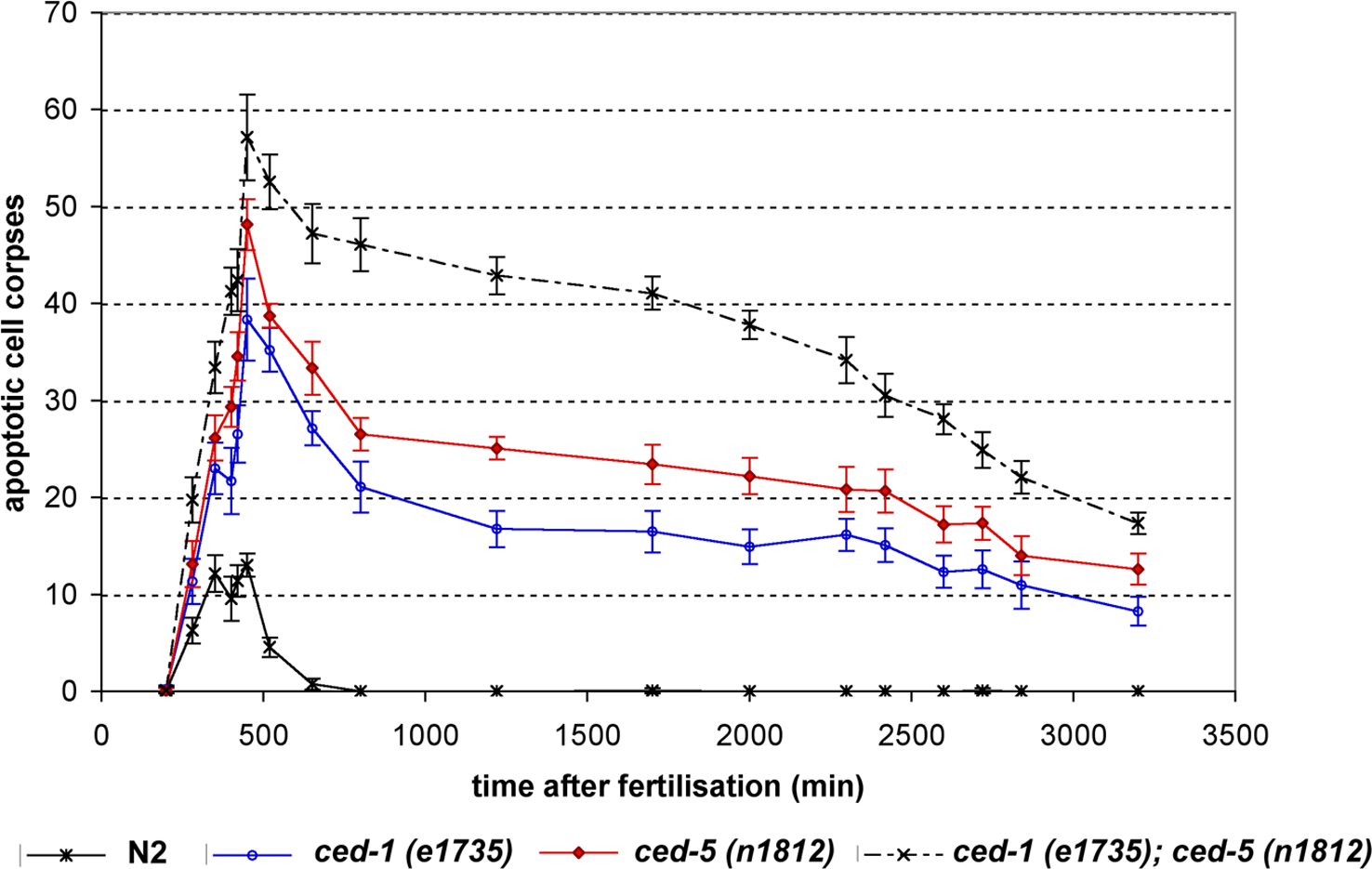

Supplement: Figure S2 — Long-term persistence of apoptotic corpses. Worms were mounted on 4% agarose pads in a drop of M9 buffer containing 30 mM NaN3 or 3–5 mM levimasole and observed by microscopy using a Leica DMRA microscope. Images were taken using an Orca ER camera using Openlab software. Worms were staged as L1s by hypochlorite treatment. Apoptotic cell corpses were identified as highly refractile disks in the germline of identically treated hermaphrodites using Nomarski optics at the time points shown in Table S2. (0.40 MB TIF) [file pbio.1000297.s002.tif]

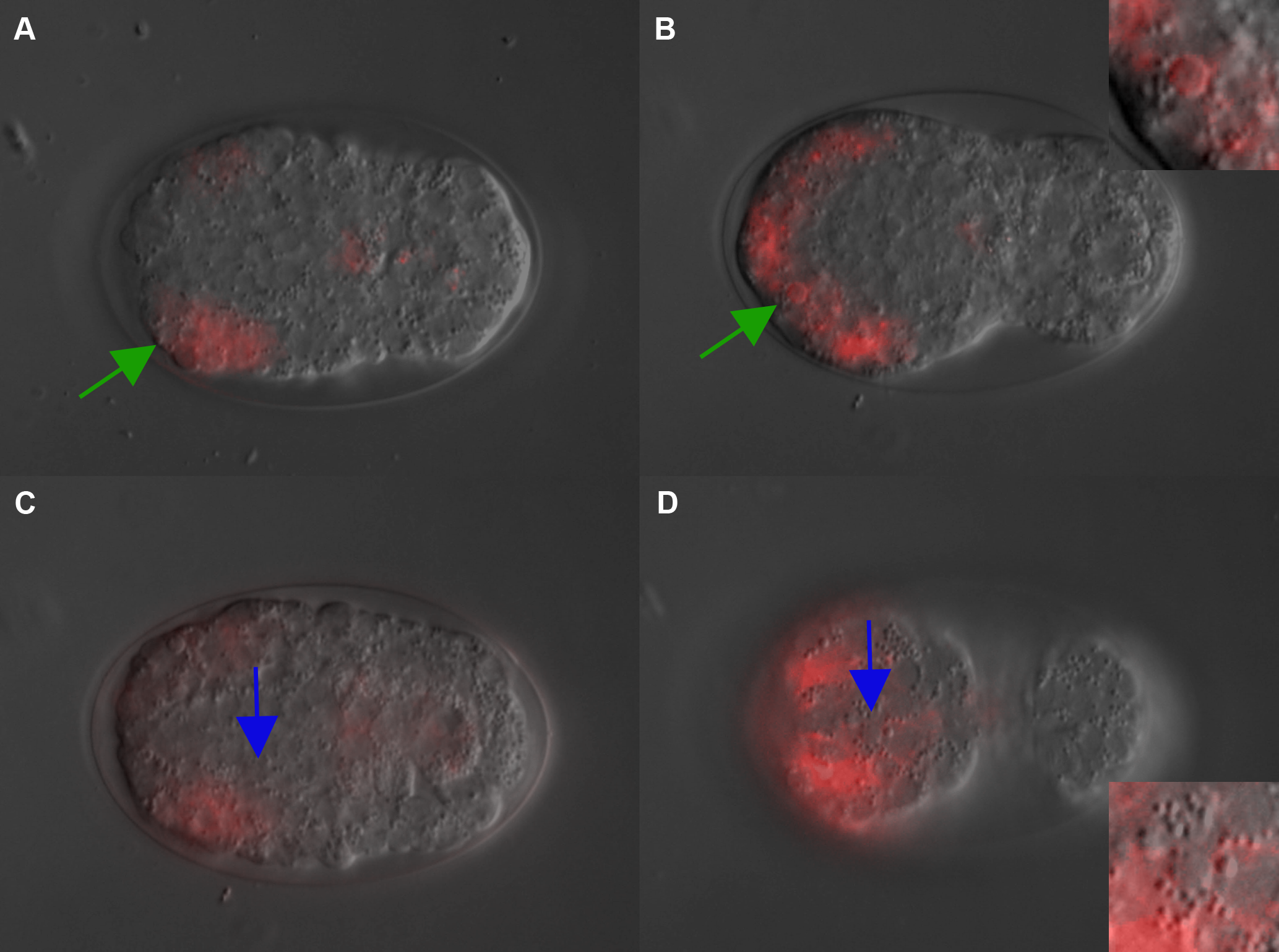

Supplement: Figure S3 — Expression of MOM-5 during engulfment of cell corpses in the embryo. The transgenic line GE4125 shows a late expression of MOM-5::mCherry, mostly in the nervous system. This mosaic expression in a wild-type background can be used to assign the localisation of the receptor to cells surrounding a cell corpse. Since all cells express MOM-5 in the embryo [24], the receptor is also present and even very much concentrated in the shrunken corpse. An example is shown in the top panels. The mother (green arrow [A]) of the corpse (green arrow) shown in (B) (inlet, top right) expresses the transgene (259 min of development). At 304 min, the corpse contains a significant amount of the fusion protein (mCherry fused to MOM-5 to replace the STOP codon) also in the interior. The mother (blue arrow [C]) of the corpse (blue arrow) shown in (D) (inlet, bottom right) does not express any visible amount of the transgene (259 min). This unlabelled corpse migrates to meet cells displaying the fluorescent signal on their surface touching the corpse (304 min). Thus the signal can be unambiguously assigned to its neighbours. The corpse is engulfed at the time the picture was taken by a cell below the corpse. These observations are consistent with our proposal of MOM-5 being involved in the engulfment of cell corpses. (2.07 MB TIF) [file pbio.1000297.s003.tif]

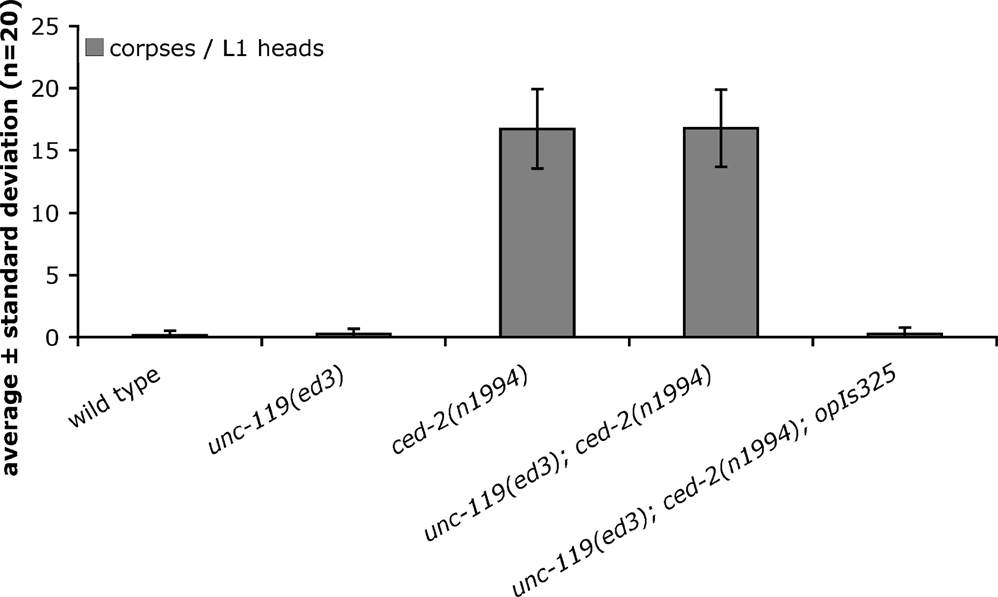

Supplement: Figure S4 — Functional rescue of the ced-2::tap construct. (0.12 MB TIF) [file pbio.1000297.s004.tif]
