# Supplementary material for: The Wnt Pathway Controls Cell Death Engulfment, Spindle Orientation, and Migration through CED-10/Rac
Source: PLoS Biol. 2010 Feb 2;8(2):e1000297. doi: 10.1371/journal.pbio.1000297 (PMC2814829; doi:10.1371/journal.pbio.1000297)
Supplement: Table S1 — The Wnt pathway affects engulfment of cell corpses in the gonad. The table shows that the classical method (Refractile corpses [DIC]) for scoring corpses by counting the corpses 12 h after the L4 moult [7] may fail to recognise an engulfment defect after a gene inactivation. This may happen if a reduction of germ cells (Progeny), as observed in mom-5(zu193), causes a reduction of cell corpses occurring in the gonad. If those, however, survive much longer because they are not engulfed, the count by the classical method may be “accidentally” normal. If corpses are followed by 4-D microscopy (Persistent corpses [4-D recording]) the engulfment defect is seen. Corpses in normal gonads are engulfed after 74.8±37.4 min (±s.d. n = 18, low: 31 min, high: 166 min). In mom-5(zu193) hermaphrodites that were in addition subjected to an RNAi against MOM-5 to deplete the maternally supplied RNA, the corpses were engulfed after 159.1±126.2 min (±s.d. n = 18, low: 32 min, high: 459 min). Considering all cell deaths, the difference is significant (t-test p<0.02, Mann-Whitney test one-sided p<0.05). We scored corpses as not properly engulfed that were not engulfed at a time equal to the wild-type mean +3 s.d. (187 min). The exact timing of events is shown in Figure 5. (0.04 MB DOC) [file pbio.1000297.s005.doc]

| **Genotype** | Refractile corpses  (DIC) | Persistent corpses  (4D-recording) | Progeny (embryos/worm) |
| --- | --- | --- | --- |
| N2 | 1.0 ± 1.3 (n=21) | 0% (n=18) | 202.2 ± 37.9 (n=12) |
| *unc-13* (*e1091*) | n.d. | 0% (n=34) | 161.4 ± 20.7 (n=12) |
| *mom-5* (*zu193*) + RNAi | 1.1 ± 1.7 (n=34) | 39% (n=18) | 17.2 ± 8.7 (n=13) |
| *ced-1* (*e1735*) | 10.0 ± 5.3 (n=10) | 57% (n=30) | 227.7 ± 14.5 (n=6) |
| *ced-5* (*n1812*) | 4.9 ± 2.6 (n=20) | 67% (n=21) | 119.2 ± 45.4 (n=6) |
| *ced-1* (*e1735*); *ced-5* (*n1812*) | 14.7 ± 5.8 (n=25) | 100% (n=33) | 94.2 ± 18.8 (n=6) |
| *wrm-1 (n1982); bar-1 (m349); hmp-2 (RNAi)* | 0.9 ± 1.7 (n=25) |  |  |
