# Supplementary material for: The Wnt Pathway Controls Cell Death Engulfment, Spindle Orientation, and Migration through CED-10/Rac
Source: PLoS Biol. 2010 Feb 2;8(2):e1000297. doi: 10.1371/journal.pbio.1000297 (PMC2814829; doi:10.1371/journal.pbio.1000297)
Supplement: Table S2 — List of developmental stages during embryogenesis and larval development analysed in Figure S2. The time after fertilisation is shown in parentheses. (0.03 MB DOC) [file pbio.1000297.s006.doc]

| **Embryogenesis** | **Larval Development** | |
| --- | --- | --- |
| ball of cells (200) | early L1 | (800) |
| tramtrack (280) | mid L1 | (1220) |
| U-view (350) | late L1 | (1700) |
| comma (400) | early L2 | (2000) |
| 1.5 fold (420) | late L2 | (2300) |
| 2 fold (450) | early L3 | (2420) |
