# Supplementary material for: The Wnt Pathway Controls Cell Death Engulfment, Spindle Orientation, and Migration through CED-10/Rac
Source: PLoS Biol. 2010 Feb 2;8(2):e1000297. doi: 10.1371/journal.pbio.1000297 (PMC2814829; doi:10.1371/journal.pbio.1000297)
Supplement: Text S1 — Biochemical analysis to demonstrate a APR-1 CED-2 interaction in vivo. A bar graph comparing the rescue of the mutant, Ced, and adult lethal phenotypes is shown in Figure S4. (0.03 MB DOC) [file pbio.1000297.s007.doc]

**Biochemical analyses**

**Co-immunoprecipitation**

Rescue strains used for co-immunoprecipitations:

*unc-119(ed3), ced-2(n1994), opIs325[P_eft-3_::tap::ced-2(cDNA)::let-858 3’UTR]* and *unc-119(ed3), alg-1(tm492), opIs205[P_eft-3_::tap::alg-1(genomic)::alg-1 3’UTR].* Both strains rescue the mutant phenotypes.; *opIs325* rescues the Ced phenotype of *ced-2* and *opIs205* rescues the adult lethal phenotype of *alg-1,* respectively (Figure S4).

**Method**

All manipulations were performed at 4°C or on ice with ice-cold buffers. 1ml of a solid worm pellet each was washed twice in icecold ddH_2_O and twice in wash buffer containing 20 mM Tris-HCl, 140 mM KCl, 1.8 mM MgCl_2_, 0.1 % NP-40, 0.02 mg/ml heparin, pH 8.0. The 1 ml worm pellets were frozen as droplets in liquid nitrogen, furthermore, 5 ml of frozen droplets of wash buffer additionally containing 1.5 mM DTT, 1 mM PMSF, 0.5 mg/ml Leupeptin, 0.8mg/ml Pepstatin, 0.2 mg/ml heparin, 20mg/ml Heparin, 100 U/ml Rnasin were added, and the mixture was broken up by a swing mill. The resulting powder was thawed, and debris as well as the fat-layer removed by spinning down the samples each 5 minutes at 3’500 (debris as pellet), 9’500 (fat-layer on top) and 12’000 rpm using a table top centrifuge at 4°C. In the mean time, IgG coupled beads (Rabbit IgG-agarose, Sigma # A2909) were washed three times in wash buffer additionally containing 0.1 mg/ml Heparin. Samples were mixed with IgG beads (1:1) and incubated for 90 min at 4°C. Beads were then washed three times in buffer containing 20 mM Tris-HCl, 140 mM KCl, 1.8 mM MgCl_2_, 10% glycerol, 1 mM DTT, 0.01% NP-40, pH 8.0. Cleavage was performed by adding 0.2 U/µl AcTEV protease (invitrogen # 12575-015) for 90 min at 25°C. Samples were then eluted using BioRad Micro Bio-Spin Chromatography columns (BioRad #732-6204) and spun down twice at 1200 rpm (table top centrifuge) in order to remove remaining beads.

**Mass spectrometric analysis**

Samples obtained by AcTEV protease cleavage were lyophilized and digested with trypsin (trypsin gold mass spectrometric grade, Promega # V5280). Peptides were desalted on ZipTip C18 pipette tips (Millipore # ZTC 18S 024) and analyzed by MALDI-TOF-MS (Ultraflex I, Bruker Daltonics) with dihydroxy benzoic acid / methylenbdiphosphonic acid (Fluka # 85707 and #64255, each 5 mg/ml in H_2_O) as matrix. Peptide mass fingerprint searches were performed with the MASCOT software (Matrixscience) ver 2.2.04 using the MSDB and NCBInr databases. Searches were restricted to viruses and *C. elegans* with a mass tolerance of 50 ppm, one missed cleavage and oxidation of methionine as variable modification. After a first search for viruses (to identify TEV protease derived peptides) a second search restricted to *C. elegans* was performed.

**MS results**

In case of the TAP::CED-2 eluate the TEV protease was identified with a MASCOT score of 107, a sequence coverage of 62% and an intensity coverage of 78%. CED-2 was identified with a MASCOT score of 78, a sequence coverage of 43% and an intensity coverage of 3.3%. There was no statistical significant evidence for the presence of APR-1 in the eluate.

For the TAP::ALG-1 eluate the TEV protease was identified with a MASCOT score of 170, a sequence coverage of 67% and an intensity coverage of 72%. Peptides for ALG-1 resulted in a sequence coverage of 4.4% and an intensity coverage of 7.8%.
